# The complete mitochondrial genome of the *Statilia maculate. *(Mantodea: Mantidae)

**DOI:** 10.1080/23802359.2016.1250134

**Published:** 2016-11-11

**Authors:** Shasha Wang, Feixia Hou, Jing Cao, Cheng Peng, Jinlin Guo

**Affiliations:** a The Ministry of Education Key Laboratory of Standardization of Chinese Herbal Medicine, Chengdu, China;; b Key Laboratory of Systematic Research, Development and Utilization of Chinese Medicine Resources in Sichuan Province, Chengdu, China;; c Key Laboratory Breeding Base of Co-founded by Sichuan Province and MOST, Pharmacy College, Chengdu University of Traditional Chinese Medicine, Chengdu, China

**Keywords:** Statilia maculate, mitochondrial genome, phylogenetic analysis

## Abstract

The complete mitochondrial genome of the *Statilia maculate* has been amplified and sequenced in this study. The mitogenome was 15,775bp long with two ribosomal RNA genes, 26 transfer RNA genes, 13 protein-coding genes, and a non-coding control region, with an A + T-rich characteristic (75.8%). Five identical tandem duplication of *trnR* were found in mitogenome of *S. maculate*, similar to the other mantis. According to the phylogenetic analysis, *S. maculate* had a closer genetic relationship with *Statilia* sp.

*Statilia maculate* (Thunberg), which belongs to *Statilia* of Mantidae of Mantodea, is a kind of important predatory insect and medicinal insect. The dry ootheca of *S. maculate*, which is named as Chang Piaoxiao, is very popular for Chinese medical resources. Chang Piaoxiao, which has significant effect on securing essence and reducing urination, enriching the kidney, and strengthening Yang-qi, is traditionally used to treat frequent micturition and seminal leakage (Committee 2015). Despite its useful applications, there is little report about *S. maculate*, except for its biological characteristic (Lin et al. 2008) and pharmacological study (Tan et al. 1997). In this study, we characterized the mitogenome sequence of *S. maculate*, and the sequence had been deposited in GenBank with accession no.KX900484.

The specimen was collected from Chengdu University of Traditional Chinese Medicine (30°58′12′′N, 103°48′36′′E) in Chengdu city, Sichuan province, China and identified as *S. maculate* based on its morphometric features and DNA barcoding technology. The sample used in this study was with Animal Ethics approval for experimentation granted by Chengdu University of Traditional Chinese Medicine, and was deposited in the herbarium of Chengdu University of Traditional Chinese Medicine (voucher ID:wjtl-2). The complete mitogenome of *S. maculate* is 15,775 bp in length, and contain 13 protein-coding genes (PCGs), two rRNA genes, 26 tRNA genes, and a non-coding control region, with an A + T-rich characteristic (75.8%). This kind of base composition bias is in agreement with other mantises with complete mitogenome reports (Tian et al. 2015; Ye et al. 2016). Eight of the 13 PCGs have ATN as the start codon, while *COI* utilize TTG, *ND1,* and *ND5* start from AAT, *ND4* translate from TCA, and *ND4L* start with TTA. Eight of the 13 PCGs terminate with incomplete stop codon (T/TA), and *COI*, *ATP8*, *ATP6*, *COIII,* and *ND6* stop with complete TAA. In 13 PCGs, the longest one is *ND5* (1695bp) and the shortest one is *ATP8* (159bp).

Five identical tandem duplication of *trnR* were found in mitogenome of *S. maculate*. This phenomenon is consistent with the other praying mantis (Ye et al. 2016), which suggested mitochondrial tRNA of Mantodea exhibited high evolutionary diversity.

The phylogenetic analysis of six Mantodea species was performed using neighbour-joining algorithm based on the whole mitogenomes in MEGA7 (Figure 1). The result further confirmed that *S. maculate* had a closer genetic relationship with *Statilia* sp. than other species of Mantodea.

**Figure 1.**
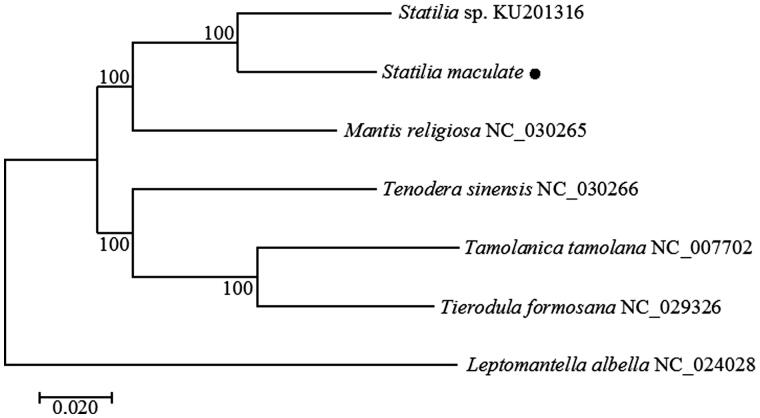
Phylogenetic tree of S. maculate and other related species in mantis based on complete mitogenome. Bootstrap values generated from 1000 replicates for NJ analysis.
